# The sympathies of the body: functional organization and neuronal differentiation in the peripheral sympathetic nervous system

**DOI:** 10.1007/s00441-021-03548-y

**Published:** 2021-11-10

**Authors:** Uwe Ernsberger, Thomas Deller, Hermann Rohrer

**Affiliations:** Institute for Clinical Neuroanatomy, Goethe University, Frankfurt/Main, Germany

**Keywords:** Noradrenergic, Cholinergic, Heartrate, Hypertension, Hydromineral, Thermogenesis, Adiposity, Immune status, Bone marrow, Skeletal health, Transdifferentiation, Synaptic organizer

## Abstract

During the last 30 years, our understanding of the development and diversification of postganglionic sympathetic neurons has dramatically increased. In parallel, the list of target structures has been critically extended from the cardiovascular system and selected glandular structures to metabolically relevant tissues such as white and brown adipose tissue, lymphoid tissues, bone, and bone marrow. A critical question now emerges for the integration of the diverse sympathetic neuron classes into neural circuits specific for these different target tissues to achieve the homeostatic regulation of the physiological ends affected.

## Introduction

According to the Danish anatomist Winslow, the great sympathetic or intercostal nerve is the principal means of bringing about the sympathies of the body (Winslow 1732; Langley 1921). In the almost 300 years since then, the progress of discoveries in the field of the autonomic nervous system, including the sympathetic nervous system, spins at an ever increasing rate. The progressing understanding of the cellular properties realized in the autonomic sympathetic and parasympathetic system (Ernsberger and Rohrer 2018), and in particular, in the postganglionic sympathetic neurons (Ernsberger et al. 2020), now becomes refined to molecular detail by single cell RNA sequencing (Furlan et al. 2016; Blum et al. 2021). Together with the studies on the integration of these neurons in autonomic reflex pathways, a comprehension of the circuits maintaining bodily homeostasis is developing (Jänig 2006). Their importance in the balancing regulation of a large number of physiological parameters as much as in the causation of a grand spectrum of autonomic disorders (Goldstein 2017, 2019) explains the ever-growing interest in autonomic neuroscience and autonomic medicine.

Here, we review key data on the importance of the sympathetic nervous system in homeostatic regulation of a range of crucial processes ranging from heartbeat and organ perfusion to water and mineral balancing, regulation of body temperature and metabolic expenditure, and the balancing of the immune system and skeletal health. We discuss the importance of target-selective sympathetic pathways to selectively regulate the different tissues involved in these processes. And we consider the developmental mechanisms for the generation of this cellular system and its integration into target-directed neural circuits.

## Key regulatory domains served by the sympathetic nervous system

From the “sympathies of the body” (Winslow 1732), the “constant milieu interieur” (Bernard 1957) the “wisdom of the body” (Cannon 1932), and the “neurobiology of homeostasis” (Jänig 2006) to the “principles of autonomic medicine” (Goldstein 2017), a comprehensive view is growing on the anatomy, physiology, pharmacology, genetics, and clinic of the autonomic nervous system. In particular, the sympathetic branch has been characterized in great detail, and its balancing interaction with the parasympathetic system is key consideration in medical physiology.

**Fig. 1:**
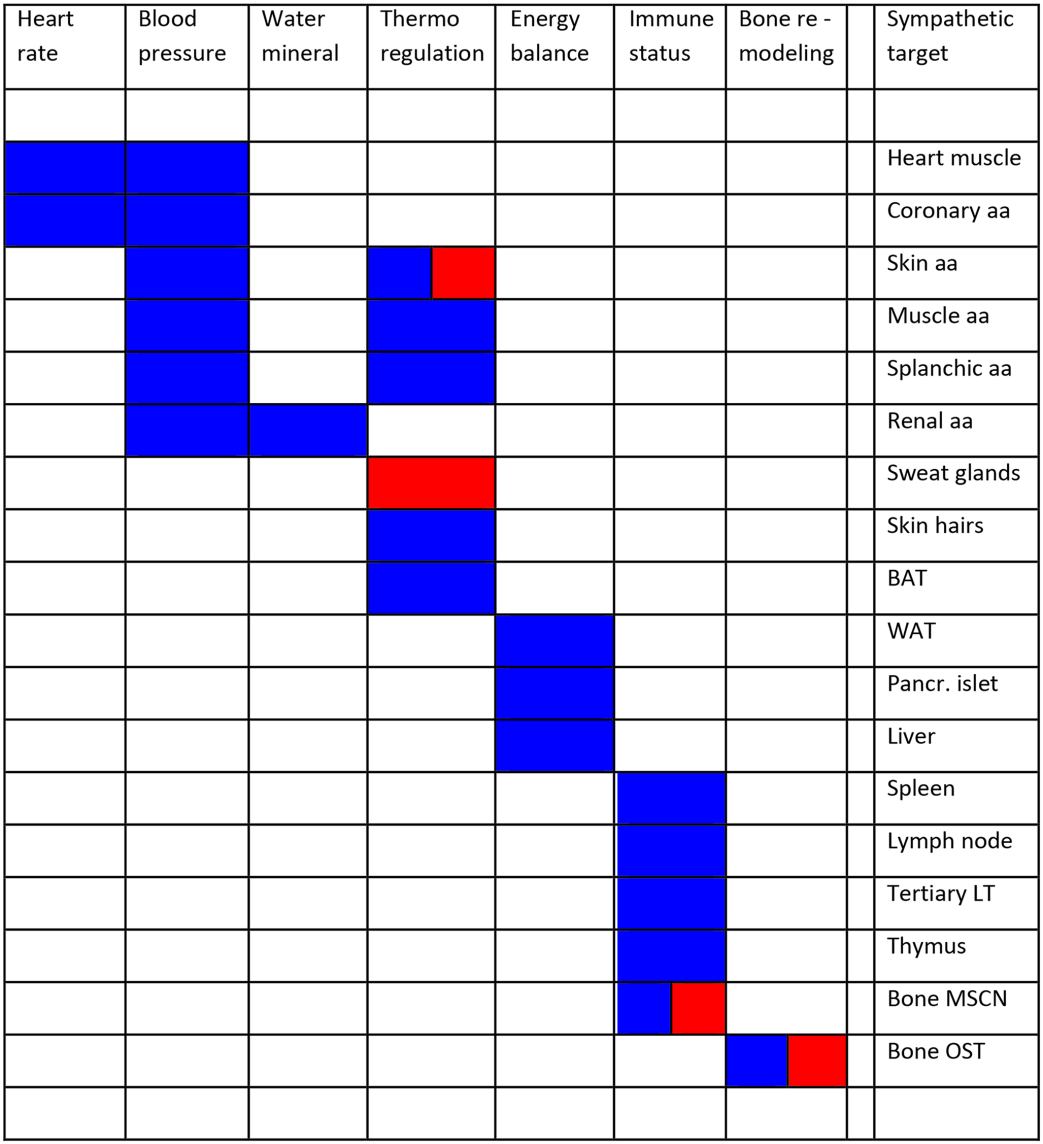
Regulatory domains in homeostatic demands, employed neurotransmitter systems and target tissues innervated by sympathetic postganglionic efferents. The figure displays the sympathetic transmitter systems employed in different target tissues. blue, noradrenergic; red, cholinergic. The data have been obtained by immunehistological detection of tyrosine hydroxylase as key marker for noradrenergic sympathetic neurons and by pharmacological discrimination of noradrenergic versus cholinergic activator or inhibitor-mediated actions. In the case of rodent sweat glands, also immunohistochemistry for choline acetyltransferase and the vesicular acetylcholine transporter have been employed in combination with retrograde labeling. Abbreviations: aa, arteries and arterioles; BAT, brown adipose tissue; MSCN: marrow stem cell niche; OST: osteoblast/osteoclast; Pancr., Pancreatic; LT: lymphatic tissue; WAT: white adipose tissue

For a long time, the sympathetic/parasympathetic antagonism on heart activity and the sympathetic control of the vascular system constituted the prime focus of this analysis. With the increased interest in metab olic control and glucose homeostasis, the growing problem of adiposity and its linkage to cardiovascular and renal disorders, as well as the tight link with immune homeostasis and the interaction between nervous and immune system, this focus has been considerably extended. A detailed knowledge of the sympathetic efferent innervation of a wide range of target tissues from blood vessels, endocrine, and adipose tissue to lymphoid tissue and bone marrow widens our view of sympathetically regulated systems. An ever-increasing body of experimental and clinical studies reveals how deeply the sympathetic nervous system (SNS) intervenes in the most diverse aspects of bodily functions (Fig. 1; Table 1).

**Table 1:** Domains of sympathetic homeostatic action, critical diseases and sympathetic target tissues

Domain	Diseases	Sympathetic target tissues
Cardiovascular	Heart failure, hypo- and hypertension	Heart and large arteries Intermediate-sized arteries Arterioles Large venous reflux vessels
Water and mineral balance	Chronic kidney disease	Kidney blood vessels Glomeruli and tubules
Thermoregulation	Hypo - and hyperthermia	Skin, muscle, and internal blood vessels Sweat glands Brown adipose tissue
Glucose and energy balance	Hypoglycemia and adiposity	Pancreatic islets White adipose tissue Hepatic blood vessels
Immune homeostasis	Immune-mediated inflammation	Bone marrow and thymus Spleen and lymph nodes Tertiary lymphatic tissues
Skeletal health	Osteoporosis	Bone and bone marrow

### Sympathetic/parasympathetic balancing in the control of heart beat and its derailment in heart failure

During the late nineteenth century, the opposing action of the vagal and sympathetic nerve supply on heart activity (von Bezold 1863; Bayliss and Starling 1892) became experimentally accessible. The observation of “antagonistic cardiac nerves” and their effect on heart rate in dogs (Samaan 1935) were complemented by studies in cats and condensed into a mathematical model of the “interrelations of vagal and accelerator effects on the cardiac rate” (Rosenblueth and Simeone 1934). Characterization of baroreceptor and chemoreceptor activation reflexes allowed to integrate the model of this antagonistic system into a homeostatic, sensory input-driven model emphasizing the antagonistic control of organ function by the sympathetic and parasympathetic system put forward by Cannon as well as nonantagonistic central control patterns as emphasized by the Brooks laboratory (Kollai and Koizumi 1979; Koizumi and Kollai 1992).

In parallel, heartrate variability (HRV) emerged as a promising tool for studying autonomic control and its central regulation (Task Force of the European Society of Cardiology and the North American Society for pacing and Electrophysiology 1996). HRV analysis is now acclaimed for its growing role as remote digital biomarker for neurovisceral integration and health in everyday life (Owens 2020) as much as for its extension to neonatal intensive care as a measure for discomfort and complications (Chiera et al. 2020).

Despite the advanced understanding of heartrate control by balancing activities of the sympathetic and parasympathetic domains of the autonomic nervous system, heart failure, different from other cardiac disorders, showed rising prevalence (Braunwald 2013; Savarese and Lund 2017). One key feature of heart failure, augmented sympathetic outflow to the heart, kidney, and muscle with chronically elevated sympathetoexcitatory reflexes (Floras 2009; Esler and Kaye 1998), demonstrates how a valuable balancing system serving homeostatic processes can be derailed to initiate a vicious circle of disease progression (Borovac et al. 2020; Zhang and Anderson 2014). Causes for this destructive readjustment can be abnormal cardiovascular reflexes associated with cardiac or renal dysfunction and central processes with chronic SNS overactivity as one of the key pathophysiological mechanisms. Thus, dysregulation of sympathetic/parasympathetic balance results in adverse alterations not only on cardiac, but also on renal or immune functions (Floras and Ponikowski 2015).

### Blood pressure control

The continuous requirement for integrated balancing action on the cardiovascular system by the autonomic nervous system during everyday life is nicely illustrated by the adjustments required within the first minute of standing up (Harms et al. 2021). The concerted action of the sympathetic and parasympathetic nervous system on heart performance and vasculature control that may be requested at intervals of less than minutes demand a balancing system of high reliability. CNS degenerative disorders frequently present with neurogenic orthostatic hypotension where these short-term autonomic control processes are insufficient (Idiaquez 2021).

The sympathetic efferents, most important for the control of mean blood pressure, constitute barosensitive units whose basal activity is directed by a network of brainstem and hypothalamic neurons (Guyenet 2006). In addition to the sensitivity of the efferent units to baroreceptor activity, the regulation of their activity state by sodium, oxygen, hormones, cytokines, osmolality, and pH nicely delineates a number of key parameters central to homeostatic control. Interestingly, this regulation also entails a respiratory modulation of sympathetic nerve activity aimed to increase the efficiency of oxygen uptake, perfusion, and cardiac performance (Molkov et al. 2014; Häbler et al. 1994).

Basic control of the blood pressure by the ANS occurs on a beat-to-beat basis typically on a timescale of seconds as required in orthostasis (Hart and Charkoudian 2014). Long-term changes in blood pressure control are typically required in changing performance, altered endurance, or with disease conditions during time. The comparison between young men and women, postmenopausal women, and old men unravels relevant differences in control mechanisms due to gender and age (Hart and Charkoudian 2014). The setpoint and key sensors of long-term blood pressure control remain an ongoing research challenge (Osborn et al. 2005; Brands 2012).

With the development of a mathematical model for blood pressure control, Guyton and colleagues provided a way to combine an analysis of the multifactorial system at a quantitative level (Guyton et al. 1972). The central importance of the renal function curve and balancing of salt and water depending on blood pressure is critical to the renal body fluid-pressure control system (Guyton 1990). Continuing exploration of this model and the obligatory role of the kidney in long-term blood pressure control and the linkage between blood pressure and sodium balance remained of vivid interest (Dorrington and Pandit 2009; Montani and Van Vliet 2009). The role of the nervous system turned into an issue of increasing importance (Osborn et al. 2009) and requested to improve classic modeling by a neurogenic model (Averina et al. 2015). In addition, gender-specific differences in long-term blood pressure control became subject of experimental and modeling analysis (Ahmed et al. 2019). Between men and women as well as within women, availability of and sensitivity to ovarian hormone exposition affect blood pressure and water regulation (Wenner and Stachenfeld 2012).

The enormous interest to understand qualitative and quantitative aspects of blood pressure control rests in the necessity to manage blood pressure in a variety of disabling and disease processes. In an incept cohort study among citizens in Framingham, Massachusetts, it was shown that high blood pressure is the most common risk factor for heart failure (Levy et al. 1996). Long-term trends in incidence and survival demonstrate gender specific differences (Levy et al. 2002). In the discussion of therapeutic strategies to prevent heart failure, intensive blood pressure lowering is a first option (Jafari et al. 2020). The major impact of the development of beta blockers to blunt noradrenergic sympathetic neurotransmission (Quirke 2006; Walker 2011) to fight hypertension (Kelly 1976; Carcel et al. 2019) points at the importance of the SNS in this regulatory system. The effect of combination therapies with beta blockers and inhibitors of the renin-angiotensin system for the treatment of heart failure highlights the importance of the SNS and the kidney (Nochioka et al. 2018; Martin et al. 2018).

### The kidney - site of water and mineral balance and effector of the sympathorenal axis in chronic disease

Renal sympathetic efferents exert a key role in water and mineral balance (Osborn et al. 2021). The regulation of renal function via glomerular filtration, sodium reabsorption, and renin release is crucial for adaptation processes at different time scales. By balancing the intake of water and electrolytes via ingestion, metabolic production of water, and excretion, an essential hydromineral homeostatic process is coordinated by aligning neural and endocrine processes in the kidney (Mecawi et al. 2015). A large degree of flexibility in the system allows individuals to operate safely despite large inter-individual differences in water uptake (Armstrong and Johnson 2018). Different from humans and other monogastric mammals, in ruminants, in addition to the kidney, the salivary glands, gastrointestinal tract, and liver essentially contribute to fluid and electrolyte homeostasis and allow existence under severe dehydration followed by rapid rehydration episodes (Silanikove 1994). Selection and breeding of domestic ruminants led to local breeds selected for their ability to dwell and withstand the challenges posed by largely diverse climatic conditions including prolonged periods of water deprivation.

The effects of renal sympathetic nerve activity (RSNA) on renal blood flow and glomerular filtration rate, tubular sodium, and water reabsorption, as well as renin release in humans, are analyzed in great detail (DiBona et al. 1997; Johns et al. 2011; Johns 2013). Increasing and decreasing RSNA show opposite effects with pathological sympathetic excitation causing sodium retention as well as blood pressure elevation (Armstrong and Johnson 2018). Its association with a series of serious comorbidities such as hypertension, heart disease, and diabetes (Belayev and Palevsky 2014; Sata et al. 2018) demonstrate how deeply the renal sympathetic efferents impact autonomic homeostasis. Blockade of sympathetic noradrenergic neurotransmission or renal denervation has shown suitable to block progression from acute kidney injury to chronic kidney disease (CKD) and to battle resistant hypertension (Noh et al. 2020; Quarti-Trevano et al. 2021).

In an extension of the model by Guyton and colleagues (see above), mechanisms of RSNA on sodium reabsorption and renin secretion were integrated (Karaaslan et al. 2005). In this manner, a model of long-term cardiovascular regulation was provided that accounts for the effect of RSNA on kidney function. This allows to simulate the consequences for RSNA and sodium excretion depending on sodium intake (Karaaslan et al. 2014).

The renocardiovascular link provided by the sympathetic efferents and the renin-angiotensin system turns out operational in essential hypertension, heart failure, CDK, and obesity/metabolic syndrome (Blankestijn et al. 2011). The identification of the renal nerve as a key therapeutic target now allows modulatory therapy, currently by nerve ablation (Schlaich et al. 2012). The observation of the increased RSNA activity associated with hypertension and of the curative effect of nerve ablation sparked an intensive debate on the question whether increased SNS activity is the dominant, sufficient, and necessary cause or may be a consequence of a diverse array of hypertensive disorders (Esler et al. 2010; Navar 2010). In a long series of comments, it appeared that the jury was hung up on the verdict (Bie and Damkjaer 2010). This discussion on the role of renal medullary perfusion continues until today (Booth et al. 2015; Bie and Evans 2017; Bądzyńska et al. 2021).

Currently the characterization of the sympathetic postganglionic system involved in the “circulatory, filtration, re-absorptive, excretory, and renin secretory contributions to overall renal function” (DiBona 2000) is incomplete. Analysis with RNA sequencing, as described below for other sympathetic neuron populations, of this diverse and functionally highly relevant sympathetic neuron population can be expected to further the comprehension of the diversity in this renal efferent innervation to judge physiological and pathophysiological roles of individual efferent neuron classes.

Taken together, the sympathorenal axis features prominently in a range of chronic diseases such as essential hypertension, heart failure, functional renal diseases, diuretic resistance, and insulin resistance accompanied by excessive central and peripheral sympathetic drive (Sobotka et al. 2011). How this relation can be quantitatively and mechanistically understood is one of the key questions in ANS function, dysfunction, and medicine.

### Temperature control: a lifesaving balancing process extending to the edge of performance and endurance limits

Maintaining a relatively elevated and stable body temperature is critical for human survival, ensuring suitable conditions for the metabolic processes and consequently for tissue and organ integrity (Tansey and Johnson 2015). This balancing endeavor is so essential that thermoregulation is frequently cited as the prototypic example of a homeostatic process achieved to a large part by autonomic nervous control of a selective set of anatomical structures (Johnson et al. 2014). Heat and cold challenges can elicit a range of physiological responses regulated by distinct sympathetic efferent pathways, including vasodilation and vasoconstriction for blood redistribution and heat convection between skin and internal organs, sweating for heat dissipation by evaporation, nonshivering thermogenesis to burn fats uncoupled from metabolic energy transfer, and piloerection important for insulation in hairy skin of many mammals.

Human thermoregulation relies largely on sympathetic sudomotor and vasomotor target pathways (Francisco and Minson 2018). Changes in skin blood flow can increase or decrease convective heat transfer from internal tissues to the periphery, thereby increasing or decreasing heat loss to the environment (Francisco and Minson 2018; Kellogg 2006; Charkoudian 2010). Sympathetic noradrenergic nerves, tonically active in a normothermic environment, mediate vasoconstriction in many vascular beds, whereas vasodilator nerves, without resting tone, mediate profound active vasodilation in glabrus skin. The understanding of the molecular processes in active cutaneous vasodilation is still incomplete but attributed to a cholinergic transmission with multiple cotransmitters (Francisco and Minson 2018). The characterization of the premotor pathways driving vasoconstriction and sweating is well underway, while the central pathways in active vasodilation are unknown (McAllen and McKinley 2018).

In humans, the thermoregulatory response with the greatest capacity for heat loss during physical activity and environmental heat exposure is sweating. It serves the bulk part of heat dissipation, and whole body sweat rate is closely linked to the evaporation requirement for heat balance (Gagnon et al. 2013). A review of comparative responses in men and women to heat stress discloses gender and age-specific differences (Kenney et al. 2014; Kenney 1985). Older people exhibit altered responses during heat stress with delayed onset of sweating and a reduction in sweat rate (Balmain et al. 2018). Whether this reduction in sweat rate is due to changes in the sweat gland or in the cholinergic sympathetic sudomotor transmission remains unclear.

In older people, there is also an impairment of heat-induced rise in skin blood flow (Balmain et al. 2018). This blunted rise in vasodilation, attributed to a compromised cholinergic sympathetic active vasodilator system, together with the reduced sweating capacity in older people, indicates that with age two sympathetic cholinergic target systems for thermoregulation become impaired. In addition to the impaired cutaneous vasodilation and sweating response, the increase in cardiac output of older individuals, a noradrenergic sympathetic target system, seems blunted as well (Kenney et al. 2014; Gravel et al. 2021).

Despite the basic thermoregulatory capacities, exercise, especially in a hot environment, can cause significant increases in internal body temperature (Kenny and McGinn 2017). Age, gender, acclimation, fitness, and chronic disease have profound consequences on the body’s ability to offset this temperature increase, and impairment may lead to sustained increases in core body temperature. Extreme conditions such as heat waves may exceed the limits of compensability in vulnerable people (Kenny and McGinn 2017; Balmain et al. 2018).

The ability to improve the regulatory competences by physical exercise and balanced heat exposure programs, in addition to dietary supplements, proves the plasticity of the autonomic homeostatic systems within personal limits. Endurance competition programs such as Iron Man triathlons in different climate zones and ultra-demanding, long-distance running competitions in the Sahara or Arctic climate zones show to which limits these regulatory systems can be stretched (Tansey and Johnson 2015; Balmain et al. 2018).

Running and swimming competitions in Arctic climate and icy water point towards another sympathetic target branch for thermoregulation. Brown fat innervation by sympathetic noradrenergic neurons is a critical effector for heat generation in brown adipose tissue by nonshivering thermogenesis (Cannon and Nedergaard 2004). In fact, brown (BAT) and white adipose tissue (WAT) are innervated by the SNS and show a wide presence of nerve terminals (Contreras et al. 2017; Blaszkiewicz et al. 2019). While postsynaptic beta 3 receptors activate lipolysis in WAT, thermogenesis is activated in BAT, such that both adipose tissues contribute to the regulation of energy balance.

Acclimation, gender, and age-specific prevalence of brown fat depots in humans indicate plasticity within the sympathetically mediated brown adipose tissue thermogenesis (Richard et al. 2012). Attenuation of the response of brown adipocytes to cold in aged humans (Graja and Schulz 2015) together with the markedly impaired reflex cutaneous vasoconstriction in response to cold stimuli in healthy older persons (Greaney et al. 2015) show that two sympathetically controlled systems to counter cold stressors are impaired with age. Taken together, sympathetic regulation during thermal stress is compromised along several sympathetic target pathways during human aging and disease and critically affects the ability to withstand both heat and cold stressors (Greaney et al. 2015).

In a comprehensive compilation of the efferent, afferent, and central neurons involved in the regulation of body temperature, a wiring diagram is developed that shows the participating sympathetic postganglionic neurons (Madden and Morrison 2019). With the neurotransmitters employed, the scheme provides part of the neurochemical code driving the regulatory system (as shown in Fig. 2 of Madden and Morrison 2019). The molecules responsible for wiring this network are to a large extent unknown. With the characterization of mouse thoracic sympathetic neurons by RNA sequencing (Furlan et al. 2016), the expression of transcripts for synaptic organizer molecules in sympathetic piloerector neurons is available (Ernsberger et al. 2020) that may be part of a neuron class-specific wiring code.

**Fig. 2:**
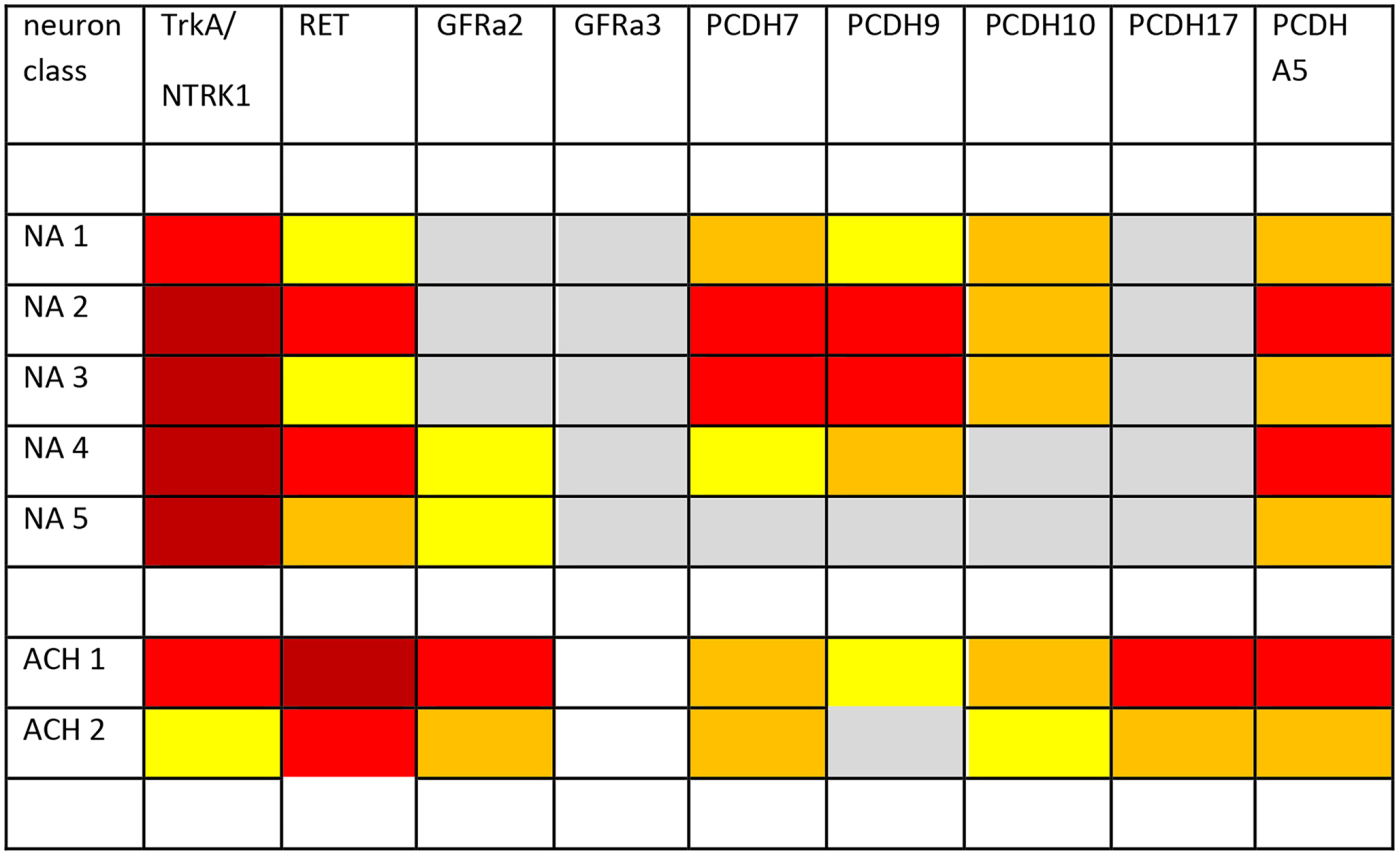
Expression of growth factor receptors and synaptic organizers of the protocadherin gene family in mouse thoracic sympathetic neuron classes. Expression level for the genes encoding the indicated receptor and organizer genes at larger than 0 (grey), 2 (yellow), 4 (orange), 8 (red), or 16 (dark red) mRNA molecules per cell. Data are taken from Furlan et al. (2016). While all noradrenergic neuron subpopulations (NA 1–5) show strong TRKA expression, the cholinergic sympathetic neurons show abundant RET expression. However, RET transcripts are detected in all, also noradrenergic sympathetic neuron classes. This observation was also made by quantitative density analysis of NBT/BCIP signals obtained from in situ hybridization in mouse cervical and thoracic sympathetic ganglia (UE, unpublished). Transcripts for GFRa receptor subunits can be detected at relatively low levels. GFRa1 and GFRa4 transcripts are not or barely observed (not displayed). GFRa3 is detected at very low levels in NA 1–5 but not in ACH 1 and ACH 2. Only GFRa2 is observed in significant levels in the cholinergic neuron populations and at low levels in noradrenergic neurons. For protocadherin transcripts, several important aspects could be derived from the single cell RNA sequencing data published by Furlan et al. (2016). Compared to other cell adhesion and synaptic organizer molecules, the PCDH gene family members are expressed very selectively in the different classes of sympathetic neurons (Ernsberger et al. 2020). For PCDH17, significant transcript levels are detected only in cholinergic neurons, not in noradrenergic neurons. The expression levels for PCDH 7, 9, 10, and A5 make it possible to distinguish all sympathetic neuron classes observed in the study. The transcripts of selected genes of the family could be detected at similar levels compared to those for TRKA and GDNF-family ligand receptors

For sympathetic neurons innervating the other efferent branches relevant for temperature regulation and metabolic homeostasis, the organizer molecules are not yet known. Since these sympathetic neuron classes are involved in the critical balancing process between food availability and temperature control (Nakamura and Nakamura 2018), further characterization of the sympathetic efferent classes and their synaptic organizer molecules will be of distinct interest.

### Glucose and metabolic homeostasis: engaging different sympathetically innervated tissues

Similar to the densely innervated brown adipose tissue involved in nonshivering thermogenesis, white adipose tissue is richly innervated by sympathetic fibers to regulate lipolysis and fat mobilization (Bartness et al. 2014). Innervation density in WAT is not as high as in BAT and detection required improved histological techniques for tissue clearing, yet roughly 90% of white adipocytes were observed to have nerve terminals nearby. In addition to adipocytes, pancreatic islets and liver are innervated by sympathetic fibers indicating a role for sympathetic efferent regulation in glucose homeostasis and lipid mobilization via different effectors (Lin et al. 2021).

Dense sympathetic and parasympathetic innervation of pancreatic islets is indicative of autonomic regulation of hormone production important for glucose homeostasis, with sympathetic activation decreasing insulin release and increasing glucagon release (Porte and Williams 1966; Ahrén et al. 1987; Lin et al. 2021). In hypoglycemic situations, this may induce a counter-regulating glucagon response (Taborsky et al. 2002). Sympathetic innervation in the liver is also involved in maintenance of glucose homeostasis by affecting net glucose uptake (Moore et al. 2012) and activating glycogenolysis (Shimazu 1996).

WAT also exerts an endocrine function and dynamically communicates with other tissues through secretion of adipokines, most importantly leptin (Lin et al. 2020). Circulating leptin inhibits food intake, depletes fat mass, and enhances glucose metabolism. Since leptin-sensitive central neuronal circuits converge onto efferent sympathetic neurons, the regulation of WAT activity by sympathetic efferents is organized in a leptin-sympathetic-leptin feedback loop (Mark et al. 2003; Kalil and Haynes 2012).

The derailment of these systems is a hallmark at the fringe of obesity and hypertension, major drivers of cardiovascular and kidney diseases (Bell and Rahmouni 2016; Hall et al. 2019). A key link between obesity and cardiovascular disease is leptin, and hyperleptinemia or leptin resistance in human obesity influence cardiovascular structure and function, inflammatory processes, and sympathetic activity that is stimulated in obese patients (Kang et al. 2020) with obvious gender differences (Shi et al. 2020). The neuroendocrine loop facilitated by adipose tissue-derived leptin and SNS-derived noradrenaline additionally engages in crosstalk with resident immune cells (Larabee et al. 2020).

### Sympathetic innervation of lymphoid tissues: harnessing the self/nonself discrimination for host defense and bodily maintenance

The perception of the autonomic nervous system as a mediator in homeostasis became considerably expanded starting in the 1960s. Immunohistochemical detection of tyrosine hydroxylase, the rate limiting enzyme for catecholamine biosynthesis, and the cell type-specific feature of noradrenergic postganglionic sympathetic neurons provided evidence for autonomic innervation in primary and secondary lymphoid tissues, notably the thymus (Felten and Felten 1989), bone marrow (Bjurholm et al. 1988), and the spleen (Fillenz 1970; Bellinger et al. 1987). Consequently, the autonomic innervation of lymphoid tissues turned into a key topic, demonstrating that all primary and secondary lymphoid tissues receive substantial sympathetic innervation (Felten and Felten 1991; Stevens-Felten and Bellinger 1997; Nance and Sanders 2007).

The presence of adrenergic receptor molecules on diverse immune cell types and the presence of sympathetic nerve fiber varicosities in primary and secondary lymphatic tissues strongly indicate classical neurotransmission (Bellinger et al. 2008). Intense sympathetic innervation is also detected in tertiary lymphoid tissue located at the meninges (Rua and McGavern 2018). Taken together, the sympathetic nervous system is now recognized as the integrative interface between two super systems, the brain, and the immune system (Elenkov et al. 2000; Wrona 2006).

Regulation of host defense by the SNS and immune homeostasis via adrenergic receptors on immune cells affects development, survival, proliferation, circulation, trafficking, and recruitment of the respective cell types (Bellinger and Lorton 2014). Consequently, this regulation impacts the activational state and functional capacity of immune cells, suggesting a critical link to the development and exacerbation of chronic immune-mediated diseases. Overall, the interaction of the nervous and the immune system are critically involved in mediating “tissue and whole body homeostasis” (Rankin and Artis 2018).

Hypertension and associated comorbidities (Lori et al. 2017) and nonpathogen-driven chronic immune-mediated diseases (Bellinger and Lorton 2018) represent examples where the homeostatic balance breaks down. In both cases, a maladaptive disease-inducing or perpetuating sympathetic response is observed and may be promoted by regional inflammation and immune dysfunction. A critical hub appears to be the spleen, where both systems are tightly linked throughout adult life. Elevated sympathetic tone, immune cell infiltration in the affected organs, and an impaired pressure/natriuresis relationship play crucial roles in hypertension and chronic kidney disease-associated homeostatic derailment (Rodriguez-Iturbe et al. 2017; Zhang et al. 2021). In neurodegenerative disease settings such as synucleinopathies or diabetic neuropathy, where also inflammatory and vascular mechanisms may converge, key autonomic disturbance may be orthostatic hypotension or neurogenic bladder and bowel dysfunction (Sasaki et al. 2020; Idiaquez et al. 2021).

### Sympathetic innervation of bone and bone marrow: regulation of skeletal health and lymphoid cell generation

Sympathetic efferent innervation to bone has major effects on bone development, remodeling, and aging on the one hand, as well as lymphoid stem cell formation and hematopoietic stem cell and progenitor cell trafficking on the other (Katayama et al. 2006; Elefteriou 2018; Fielding and Méndez-Ferrer 2020; Tomlinson et al. 2020). Thus, sympathetic innervation to the bone and bone marrow contributes to skeletal health and immune homeostasis.

Both the noradrenergic and the cholinergic neurotransmitter systems are employed by the sympathetic postganglionic system to the bone. With diurnal rhythmic activities of sympathetic noradrenergic as well as cholinergic efferents, migration of hematopoietic stem cells and leukocytes from bone marrow are regulated (del Toro and Méndez-Ferrer 2013; García-García et al. 2019). In addition, together with complex hormonal changes during pregnancy and lactation, sympathetic efferent outflow orchestrates bone remodeling across longer timescales (Winter et al. 2020). Finally, alterations of sympathetic system activity with aging as observed by HRV analysis in elderly (Nicolini et al. 2012) and the age-related decline to frailty and late-life vulnerability (Walston 2015) disclose plasticity on long biological timescales (Otto et al. 2020). In disease, autonomic nervous dysfunction may link disruptions in bone remodeling and immune status as observed in osteoporosis in multiple sclerosis (Sternberg 2018).

With the demonstration of leptin as a powerful inhibitor of bone formation (Ducy et al. 2000) and evidence from DBH-deficient mice and pharmacological intervention that the leptin action on bone formation occurs via the sympathetic nervous system (Takeda et al. 2002), the noradrenergic sympathetic pathway regulating bone homeostasis became a key topic. A critical finding was the demonstration that among the two phases of bone remodeling, bone resorption, and bone formation, the former is favored by sympathetic nervous activity due to promoting an osteoclast differentiation pathway through noradrenergic transmission (Elefteriou et al. 2005). A sympathetic dominance during aging may come with disturbed bone remodeling observed in osteoporosis (Elefteriou 2018).

With the demonstration of cholinergic differentiation in initially noradrenergic sympathetic neurons innervating bone (Asmus et al. 2000), requirement for GFRalpha-2 for cholinergic sympathetic fiber innervation at the periosteum (Hiltunen and Airaksinen 2004), the differences in noradrenergic and cholinergic pathways to different bone envelopes (Bataille et al. 2012), and the involvement of cholinergic sympathetic activity in the regulation of hematopoietic stem cell and leukocyte migration (García-García et al. 2019), the generation and function of these cholinergic sympathetic neurons becomes a new focus of interest.

Thus, the diverse sympathetic innervation to bone and bone marrow and consequently its involvement in the regulation of skeletal health on the one hand and hematopoietic stem cell dynamics on the other prompt the questions for the generation of the neuronal diversity involved and for the wiring of the diverse sympathetic neuron types to their target cells as well as their preganglionic input.

### The domain specific access to sympathetic targets: the concept of target-specific sympathetic pathways

Sympathetic efferent activity shows parallel responses to many stimuli but differences may occur according to targets, such as the heart and kidney, and the stimulation setting (Guyenet 2006; Jänig and McLachlan 1992). Differential activity patterns are best shown by recordings from selected nerve filaments supplying different targets (Esler 2010). In the period from 1970 to 2010, a large amount of observations on differential regulation of the sympathetic activity destined towards distinct target tissues was compiled, and different or even opposite changes in the sympathetic outflow to different domains of the cardiovascular system in response to thermal stimuli represent a critical observation (Jänig and Häbler 2000; Iriki and Simon 2012).

Consequently, the efferent action of the sympathetic nervous system to its highly diverse target organs and the need to appropriately balance a variety of body functions and parameters require a specified signaling. The assumption that indices of SNS drive to the heart, skeletal muscle, or skin can be generalized in the line of thinking of Cannon’s “fight and flight response” with a uniformly activated SNS can be misleading (Bartness et al. 2014).

This change in the perception of the patterning of sympathetic outflow is nicely captured in a summary by Morrison: “ ... the early views of the sympathetic nervous system as a monolithic effector activated globally in situations requiring a rapid and aggressive response to life-threatening danger have been eclipsed by an organizational model featuring an extensive array of functionally specific output channels that can be simultaneously activated or inhibited in combinations ...” (Morrison 2001). New data sets on single cell transcriptomes have the potential to unravel the organizing molecules which may be involved in the generation of these specific output channels.

## Domains of progress in understanding the generation, specification, and connectivity of sympathetic neuron classes

With their landmark study on the growth stimulatory effects of mouse sarcoma on the sensory and sympathetic nervous system in chick embryos (Levi-Montalcini and Hamburger 1951), Levi-Montalcini and Hamburger laid out a key concept in molecular developmental neuroscience (Levi-Montalcini 1987). The working of nerve growth factor (NGF) on sympathetic neurons fits perfectly into the frame of the neurotrophic theory (Oppenheim 1989) stating that a set of neurons in a population will die because trophic factors are available only in limiting amounts, in case of NGF corresponding to the size of the innervated target field. Thus, early developmental neuroscience was dominated by a concept of size adjustment between neuron populations and corresponding target field.

Another important move forward was the characterization of a set of growth factors involved in differentiation and patterning and their induction of neuronal and noradrenergic differentiation in neural crest-derived progenitors to develop into sympathetic neurons (Rohrer 2011). With the induction by these growth factors of a transcription factor network with cross-regulatory properties dominated by the PHOX2b master regulator (Pattyn et al. 1999), the development of the sympathetic autonomic cell lineage became understandable in molecular terms. At the same time, failures in proliferative control by this network make this system prone to tumorigenesis and explain derailment of sympathetic neuron differentiation to childhood tumor formation.

This progress is now accelerated by a major technical advancement, single cell RNA sequencing. The identification and characterization of sympathetic preganglionic and postganglionic neurons by their full transcriptome allows specific molecularly guided characterization of the diverse autonomic nerve cell classes. Developmental analysis is performed by the combination of RNA sequencing and the analysis of genetically labeled neuron classes (Furlan et al. 2016). Moreover, information contained in these single cell transcriptomes on gene products involved in electrical activity (ion channels and transmitter receptors) or synapse formation and synaptic information propagation (synaptic proteins, synaptic organizers, and cell adhesion molecules) promise the study of generation and function of target-specific efferent pathways.

### Neural crest precursor migration and initial sympathetic differentiation: generation of mixed noradrenergic/cholinergic neuroblasts by a PHOX2b-directed transcription network prone to malignancy

The progenitor cells destined to form neurons in the sympathetic ganglia are derived from the neural crest, a transient structure at the dorsal tips of the closing neural fold (Kalcheim 2018). The cells undergo an epithelial-to-mesenchymal transition to then follow ventrally directed migration routes towards the sites of initial differentiation in primary sympathetic ganglia adjacent to the aorta (Bronner-Fraser 1995; Kulesa et al. 2009). Upon arrival in the vicinity of the dorsal aorta, differentiation events result in the coordinated expression of noradrenergic as well as neuronal properties (Rohrer 2011; Ernsberger 2000).

Growth factors critically involved in this inductive process are bone morphogenetic proteins 4 and 7 that become expressed in the wall of the developing aorta (Reissmann et al. 1996; Shah et al. 1996; Schneider et al. 1999). In addition, a notochord-derived signal was found important in early experiments in the chick embryo (Groves et al. 1995). Importantly, BMPs and notochord-derived sonic hedgehog have shown to be instrumental in the differentiation of sympathetic neurons from human pluripotent stem cells (Huang et al. 2016; Oh et al. 2016; Frith and Tsakiridis 2019).

The growth factor-induced differentiation process is driven by a transcription factor (TF) network critically involving PHOX2b, HAND1 and HAND2, and GATA2 and GATA3, linked by cross regulatory capacity (Rohrer 2011; Ernsberger and Rohrer 2018; Ernsberger et al. 2020). Notably, PHOX2b is required for initiation of the noradrenergic and neuronal differentiation process (Pattyn et al. 1999) and for maintenance of noradrenergic marker expression in differentiated neurons (Coppola et al. 2010). A comprehensive outline of expression and function of these transcription factors in sympathetic neuron development and their importance in the regulation of progenitor and neuroblast proliferation and differentiation is provided by studies from several laboratories in different mammalian and avian species (Ernsberger and Rohrer 2018).

Importantly, in noradrenergic neuroblastoma cell lines, super enhancers that govern gene expression programs are associated with gene loci for the TFs PhOX2a and 2b, HAND2, and GATA2 and 3 (Boeva et al. 2017; van Groningen et al. 2017). Deregulation of embryonic neural crest (NC) development results in this NC-derived pediatric malignancy (Tomolonis et al. 2017; Johnsen et al. 2019). Already more than a decade ago, the importance of PHOX2b and Delta-Notch pathway has been observed in a number of familial neuroblastoma (van Limpt et al. 2005; Raabe et al. 2008). Analysis of the action of neuroblastoma PHOX2b variants in developing sympathetic neurons demonstrated their stimulatory effect on neuroblast proliferation combined with dedifferentiation indicating routes to malignancy modulated by PHOX2b in combination with HAND2 (Reiff et al. 2010). Studies for other transcription factors of the network directing sympathetic neuron differentiation and their role in neuroblastoma exist for GATA3 (Almutairi 2019; Moriguchi 2021) and ASCL1 (Wang et al. 2019; Ali et al. 2020).

Apart from their involvement in the formation of pediatric solid tumors (Capasso et al. 2020; Pudela et al. 2020), PHOX2B mutations leading to polyalanin-expansion cause congenital central hypoventilation syndrome (CCHS) (Keywan et al. 2021). In addition to CCHS PHOX2B variants define a broad phenotypic spectrum, including Hirschsprung disease, apparent life-threatening event (ALTE) and sudden infant death syndrome (SIDS) (Bachetti and Ceccherini 2020). These observations demonstrate the key strategic role of PHOX2b-guided developmental processes in critical life-sustaining functions.

Segregation of noradrenergic neuronal and adrenergic endocrine sympatho-adrenal lineages occurs early during development, with endocrine cells in the adrenal medulla generated from Schwann cell precursors as determined in mouse and human embryogenesis (Furlan et al. 2017; Jansky et al. 2021; Kameneva et al. 2021). Minor differentiation capacity from Schwann cell precursors to sympathetic neuroblasts can be detected in human sympathoadrenal development as observed by single cell RNA sequencing (Kastriti et al. 2019; Kameneva et al. 2021).

During initial differentiation in the sympathetic neuron lineage, cholinergic properties are detectable soon after noradrenergic induction in birds and mammals (Ernsberger et al. 1997; Huber and Ernsberger 2006; Furlan et al. 2013; Huang et al. 2013). These neurons are considered to be of mixed neurotransmitter phenotype. Subsequently, a segregation process starts which rapidly leads to the restriction of the noradrenergic or cholinergic transmitter synthesis and vesicular storage capacities in distinct sympathetic postganglionic neurons innervating specific target tissues.

Taken together, neural crest-derived progenitors migrating through somatic mesenchyme towards the aorta are induced to engage in an orchestrated differentiation process by BMPs and SHH, growth factors involved in dorsal-ventral embryonic patterning. Among the first markers indicative of this differentiation process are a set of TFs dominated by PHOX2b, GATA3, and HAND1 and 2 followed by the noradrenergic neurotransmitter phenotypic enzymes and transporters. Surprisingly, cholinergic properties are detectable only with short delay, producing early neurons of mixed transmitter phenotype. A peculiarity in sympathetic neuron development is the maintenance of proliferative activity after initial differentiation (Rothman et al. 1978; Rohrer and Thoenen 1987). For the differentiation process proper, precursors or neuroblasts exit the cell cycle to then reenter proliferative activity (Gonsalvez et al. 2013). The generation of the pediatric tumor neuroblastoma from sympathoadrenal progenitors and the importance of TFs of the sympathetic neuron differentiation pathway in tumor generation point towards a soft spot in the system.

### The generation of sympathetic neuron diversity: a complicated process composed of embryonic target-independent and postnatal target-dependent events

The generation of the mature neuron classes from the early, often mixed noradrenergic and cholinergic neuroblasts is a process that rapidly commences embryonically after initial differentiation and extends well into the postnatal period (Cane and Anderson 2009; Ernsberger et al. 2020). Control of this process by growth factors and transcriptional regulators became a focus of research (Rohrer 2011) since the trans-differentiation of sympathetic neurons innervating rodent sweat glands during early postnatal maturation was described (Francis and Landis 1999). Consequently, the target-dependent determination of the neurotransmitter phenotype in sympathetic ganglia became a key topic in developmental neurobiology (Schotzinger et al. 1994), while target-independent differentiation processes remained neglected for some time.

PHOX2b, critical for the initial differentiation process, is required for induction of both the noradrenergic (Pattyn et al. 1999) as well as the cholinergic properties (Huber and Ernsberger 2006) as demonstrated in mouse embryos. RET signaling is essential for the maintenance of cholinergic marker expression, choline acetyltransferase (CHAT), as well has the vesicular acetylcholine transporter (VACHT), throughout embryogenesis but not for its initial induction (Burau et al. 2004; Furlan et al. 2013). Mutational inactivation in mice leads to the loss of cholinergic marker expression, without apparent effect on the expression of noradrenergic properties such as TH and DBH. However, target tissues innervated by these embryonically cholinergic sympathetic neurons and their postnatal fate are not known. Many cholinergic sympathetic neurons are only generated postnatally by trans-differentiation from noradrenergic neurons.

The embryonic segregation of functional marker gene expression from mixed noradrenergic/cholinergic neuroblasts to mature neurons with either noradrenergic or cholinergic neurotransmitter phenotype was studied in detail in chick and mouse embryos. Experimental analysis in rodents documented the restriction of the enzymes and the vesicular neurotransmitter transporters for the noradrenergic and cholinergic neuron phenotypes at mid-embryonic stages before innervation of the final targets (Ernsberger et al. 1997; Huber and Ernsberger 2006; Furlan et al. 2013). This developmental transition coincided with the appearance of TRK A and the disappearance of RET and TRK C expression in the majority of sympathetic neurons (Fagan et al. 1996; Furlan et al. 2013). The homeobox transcription factor 1 (HMX1) is required for the TRK A expression as well as the RET suppression (Furlan et al. 2013).

Different from the embryonic maintenance of cholinergic properties by RET signaling, the postnatal trans-differentiation of noradrenergic sympathetic neurons in mammalian sweat glands towards a cholinergic transmitter phenotype is not mediated by RET but relies on gp130 signaling as shown in rat and mice (Habecker et al. 1997; Stanke et al. 2006). Growth factors binding to this receptor system also mediate sweat gland maturation in humans as inferred from the cold-induced sweating syndrome in homozygous mutations of the receptor (Melone et al. 2014; Oxford 2016). The postnatal trans-differentiation of initially noradrenergic neurons to cholinergic neurons innervating bone (Asmus et al. 2000) molecularly appears to rely on a different signaling system (Asmus et al. 2001) by a yet undefined growth factor.

Characterization of the neuron classes in adult mouse stellate and thoracic sympathetic ganglia by single cell RNA sequencing identified a range of molecularly distinct cell types (Furlan et al. 2016). This identified seven classes of efferent neurons, five with noradrenergic and two with cholinergic neurotransmitter characteristics which also show distinct growth factor receptor expression patterns. Two of these classes could be assigned to erector muscle neurons innervating piloerectors and nipple erector muscles which in the mature state express TRK A as well as RET. Organogenesis in the nipple and piloerector muscles occurs during the first postnatal days together with induction of RET expression and a marked soma size increase in the innervating neurons, indicating postnatal identity acquisition similar to sweat gland and bone innervating neurons, albeit without a change in the neurotransmitter phenotype. Differences in GFR alpha expression in cells destined to become nipple or piloerectors at an early postnatal time point preceding RET expression and final differentiation suggest the diversification of RET and GFR alpha expression as one critical path of sympathetic neuron differentiation next to the neurotransmitter phenotype.

The combination of genetic cell lineage tracing and single cell RNA sequencing now allows to considerably sharpen our idea about the nature of efferent sympathetic neuron classes. Data sets generated by this method also allow detailed analysis on the expression of voltage-gated ion channels, neurotransmitter receptors, and synaptic proteins to provide a picture of the information processing and propagating characteristics of the cell classes (Ernsberger et al. 2017). As detailed below, this method also provides access to data that may guide our thinking about the nature and development of target-specific sympathetic circuits.

### Formation of synaptic connections between pre- and postganglionic neurons in sympathetic ganglia: final step in the formation of target specific pathways

The sympathetic ganglia are the relay sites where the central input undergoes a final processing operation when transmitted from the preganglionic neurons and translated to the efferent activity pattern recordable from the postganglionic neurons. In a classical set of electrophysiological analysis on superior cervical ganglion neurons from guinea pigs, estimates were derived on the numbers of preganglionics terminating onto individual postganglionic neurons which number to a minimum of 10 preganglionic neurons from four adjacent spinal cord segments converging onto one postganglionic neuron (Njå and Purves 1977; Purves and Wigston 1983). At the same time, one preganglionic neuron is estimated to contact approximately 50–200 postganglionic neurons, indicating the transmission of the activity status of one preganglionic neuron to a relatively large number of postganglionic neurons. Expectedly, these numbers differ between mammalian species (Lichtman and Purves 1980).

Importantly, there is a paring down in the number and distribution across spinal cord segments of preganglionic neurons converging onto one postganglionic cell during early postnatal life (Lichtman and Purves 1980). The observation that target deprivation or application of an NGF antiserum results in a reduced ganglionic transmission, and the number of synapse profiles on individual postganglionic neurons, which can be prevented by NGF administration (Njå and Purves 1978), demonstrates the importance of target and target-derived factors on the innervation of postganglionic neurons by preganglionic fibers. It is reasonable to assume that the postnatal reduction in the convergence of preganglionic inputs to postganglionic sympathetic units and the regulation of the degree of convergence by the target share similar mechanisms.

The impact of nerve growth factor on the formation of synaptic specializations was confirmed in NGF mutant mice, where neuronal loss by cell death was prevented due to an additional BAX mutation (Sharma et al. 2010). NGF acts via binding to TRK A, and its retrograde endosome transport into the dendrites is required for maintenance of a normal synaptic bouton complement (Lehigh et al. 2017). This process mediated by the target-derived growth factor NGF is complemented by preganglionic neuron-mediated communication via the neurotrophin BDNF that promotes synapse formation (Causing et al. 1997) which, in addition, is involved in axon pruning (Singh et al. 2008). Thus, 2 neurotrophins have been shown to be involved in the formation and maturation of the synaptic complement connecting preganglionic and postganglionic sympathetic neurons. The factors wiring different sympathetic target pathways in sympathetic ganglia by favoring the formation of synapses between selected preganglionic neurons and specific classes of postganglionic nerve cells or by removing connections between inappropriate neuron classes are unknown so far (Ernsberger and Rohrer 2018).

With the availability of the complete genome-wide RNA profiles of large numbers of sympathetic neurons, the search for neuron class-specific features opens new alleys to address the question for the molecular mediators of sympathetic pathway delineation (Table 2). In a large sample of mouse stellate and upper thoracic sympathetic neurons (Furlan et al. 2016), the characterization of seven classes of noradrenergic and cholinergic sympathetic neurons allowed the identification of cell adhesion and synaptic organizer molecules with remarkable neuron class-specific expression patterns (Ernsberger et al. 2020; Fig. 2). While the mRNA expression levels for cell adhesion molecules like NCAM1 or L1CAM barely differ between the different sympathetic neuron classes, protocadherine 7, 9, and 17 mRNA levels differ between the neuron classes by more than tenfold, for PCDH7 to an extent similar to the highly specific marker genes vesicular monoamine transporter 2 and acetylcholine transporter.

**Table 2:** Characterization of the neuronal elements and their connectivity involved in the formation of target-selective pathways in sympathetic ganglia

Preganglionic input	Divergence/convergence	Postganglionic output
16 preganglionic classes in mouse spinal cord (Blum et al. 2021)	1 preggl. neuron contacts 50 to 200 postggl cells; 1 postggl. neuron receives minimal 10 preggl inputs in guinea pig SCG (Njå and Purves 1977; Purves and Wigston 1983)	7 postganglionic classes NA 1 to 5, ACH 1, and ACH 2 in mouse sympathetic ganglia (Furlan et al. 2016)
	1 postggl neuron receives 6-7 preggl inputs in adult; 1 postggl n. receives more than 11 preggl inputs in young hamster SCG (Lichtman and Purves 1980)	

Characterization of the preganglionic and postganglionic sympathetic neuron classes in mice was performed by single cell RNA sequencing. The number of preganglionic neuron classes identified by whole genome gene expression patterns from ChAT-positive spinal cord cells located at cervical to sacral levels exceeds the number of postganglionic neuron classes identified in the same manner from stellate and upper thoracic sympathetic ganglion cells

The characterization of the connectivity between pre- (preggl) and postganglionic (postggl) neurons in the guinea pig and hamster superior cervical ganglia was performed by intracellular recording. These studies show that individual preganglionic neurons innervate relatively large numbers of postganglionic cells. Individual postganglionic cells receive innervation by a comparatively small set of preganglionic neurons. This postganglionic innervation seems to become refined during development from young to mature animals

In studies on genome-wide RNA profiles of mouse CHAT-positive spinal cord neurons including preganglionic sympathetic neurons, 16 classes of molecularly distinct preganglionic neuron types could be distinguished (Blum et al. 2021; Alkaslasi et al. 2021). Importantly, histograms of the relative mRNA expression levels of PCDH 7 versus 9 demarcated distinct relative expression quantities for each of the preganglionic neuron classes (http://spinalcordatlas.org). Since this molecular class has been involved in multiple steps of neural circuit formation based on homophilic interactions (Kim et al. 2011; Peek et al. 2017), its role in the formation of synaptic connections between preganglionic and postganglionic sympathetic neurons deserves experimental attention. The contribution to the etiology of neurodevelopmental disorders (Mancini et al. 2020) may not be restricted to higher-order cognitive disturbances.

## Concluding outlook

The amazing advancements in understanding of the cellular structure and developmental generation of the sympathetic nervous system now allows to investigate the generation of target-specific efferent pathways. The identification of the molecular players regulating formation of the appropriate synaptic connections in sympathetic ganglia is one of the most exciting imminent questions regarding the development of the autonomic nervous system. At the same time, autonomic dysfunctions and alterations in the development of the sympathetic nervous system remain an immense or even increasing clinical challenge.

The urgent need for new therapies for childhood tumors (Zafar et al. 2021) puts emphasis on the improved understanding of the transition from cycling progenitors to mitotic inactive cells. This incompletely understood developmental progression in neural crest and sympathetic progenitors and neuroblasts holds a range of pressing questions. On the one hand, the unfolding of nuclear chromatin during segregation of noradrenergic and cholinergic neurotransmitter phenotypes together with the acquisition of a pan-neuronal transcriptome is still not fully understood. In addition and maybe even more important, the regulation of the overall mitotic cycle number in sympathetic neuroblasts and the molecular triggers for final mitotic withdrawal are still open (Chan et al. 2018). Rapidly progressing investigation in this field is expected to provide answer to these fundamental questions and will likely yield improved neuroblastoma therapy options (Durinck and Speleman 2018; Fetahu and Taschner-Mandl 2021).

The diverse spectrum of disease-associated autonomic dysfunctions is the subject of autonomic medicine (Goldstein 2017). While the challenges in many areas are increasing, new diagnostic and therapeutic options become apparent. Progress in peripheral neural interface technology will improve bioelectronic neuromodulation and will refine this avenue of fighting defined, organ-selective dysregulation (Cho et al. 2020). Next to direct neuromodulatory intervention, electronic health applications will restructure the monitoring and surveillance of individual health conditions (Scholz et al. 2021; Cheshire et al. 2020). The possibility of self-monitoring outside the hospital will change access of patients to their diseases and will open a new chapter in personalized medicine.

## Abbreviations

ALTE: Apparent life-threatening event
ANS: Autonomic nervous system
BAT: Brown adipose tissue
ASCL1: Achaete-scute family bHLH transcription factor 1
BAX: BCL2 associated X, apoptosis regulator
BDNF: Brain-derived neurotrophic factor
BMP: Bone morphogenetic protein
CCHS: Congenital central hypoventilation syndrome
CHAT: Choline acetyltransferase
CKD: Chronic kidney disease
CNS: Central nervous system
DBH: Dopamine beta hydroxylase
GATA: GATA binding protein
GFR: Glial cell line-derived neurotrophic factor receptor
GP 130: Interleukin 6 signal transducer
HAND: Heart and neural crest derivatives expressed
HRV: Heartrate variability
L1CAM: L1 cell adhesion molecule
NCAM: Neural cell adhesion molecule
NGF: Nerve growth factor
PCDH: Protocadherin
PHOX2B: Paired-like homeobox transcription factor 2b
RET: Ret proto-oncogene
RSNA: Renal sympathetic nerve activity
SCG: Superior cervical ganglion
SHH: Sonic hedgehog
SIDS: Sudden infant death syndrome
SNS: Sympathetic nervous system
TH: Tyrosine hydroxylase
TF: Transcription factor
TRK: Neurotrophic tyrosine kinase receptor
VACHT: Vesicular acetylcholine transporter
WAT: White adipose tissue
